# Permutation Entropy-Based Analysis of Temperature Complexity Spatial-Temporal Variation and Its Driving Factors in China

**DOI:** 10.3390/e21101001

**Published:** 2019-10-13

**Authors:** Ting Zhang, Changxiu Cheng, Peichao Gao

**Affiliations:** 1 State Key Laboratory of Earth Surface Processes and Resource Ecology, Beijing Normal University, Beijing 100875, China; zhangting_bnu@mail.bnu.edu.cn (T.Z.); gaopc@bnu.edu.cn (P.G.); 2 Faculty of Geographical Science, Beijing Normal University, Beijing 100875, China; 3 Center for Geodata and Analysis, Beijing Normal University, Beijing 100875, China

**Keywords:** permutation entropy, air temperature fluctuation complexity, spatial variation, temporal variation, driving factors

## Abstract

Air temperature fluctuation complexity (TFC) describes the uncertainty of temperature changes. The analysis of its spatial and temporal variation is of great significance to evaluate prediction uncertainty of the regional temperature trends and the climate change. In this study, annual-TFC from 1979–2017 and seasonal-TFC from 1983–2017 in China were calculated by permutation entropy (PE). Their temporal trend is described by the Mann-Kendall method. Driving factors of their spatial variations are explored through GeoDetector. The results show that: (1). TFC shows a downward trend generally, with obvious time variation. (2). The spatial variation of TFC is mainly manifested in the differences among the five sub-regions in China. There is low uncertainty in the short-term temperature trends in the northwest and southeast. The northeastern and southwestern regions show high uncertainties. TFC in the central region is moderate. (3). The vegetation is the main factor of spatial variation, followed by the climate and altitude, and the latitude and terrain display the lowest impact. The interactions of vegetation-altitude, vegetation-climate and altitude-latitude can interpret more than 50% of the spatial variations. These results provide insights into causes and mechanisms of the complexity of the climate system. They can help to determine the influencing process of various factors.

## 1. Introduction

The climate system is complex, with nonlinear, multilevel and forced dissipation characteristics [1]. Its complexity characteristics have attracted more and more researchers in previous studies e.g., [2,3,4]. The measurement of such intrinsic complexity has theoretical significance and practical value for climate system research. The climate system keeps on changing and evolving, and temperature is one of the most sensitive elements in climate system [5]. In the traditional research, meteorologists mostly use the average [6,7] or probability density function [8,9] of specific index to analyze the temperature spatiotemporal variation. However, the description of temperature fluctuation complexity (TFC) is also important, which is of great significance to evaluate the predictability of climate change [10]. At the same time, TFC also provides a new perspective and information dimension to describe the spatial and temporal distribution of the air temperature. Considering the complexity of the climate system, it is reasonable and feasible to use the concept of uncertainty in complexity science to quantify TFC, and then to reveal TFC spatial and temporal variation. By measuring the uncertainty or randomness of temperature changes, the analysis of TFC can quantify the complexity of the climate system [11].

With the improvement of human cognitive abilities, complexity science is playing an increasingly important role in the study of climate system and geography [12,13,14,15,16,17]. Various methods have sprung up to describe the internal complexity feature of the climate system which are neglect in traditional statistics [18,19]. Among them, permutation entropy (PE) reflects the system dynamic complexity [18]. Compared with other algorithms (like approximation entropy and Lyapunov exponents), the PE algorithm has better anti-noise ability and can detect uncertainties in complex systems by considering the temporal order of time series [20,21]. It also has other advantages including clear physical concepts and simple calculation process [21,22]. Meanwhile, this method has been widely used to evaluate the uncertainty of sequence trends in terms of hydrological system [23], stock market in economics [24], medical signal analysis [25], mechanical fault diagnosis [26] and so on. Therefore, PE is selected to measure the complexity and predictability of the local temperature changes in our research. In the areas with high PE, the uncertainty of temperature changes is large, and its temperature tendency is hard to be predicted accurately; whereas in places with low PE, there is low uncertainty and high predictability of its temperature change.

In recent studies, some researchers have applied PE to reveal the temperature fluctuation complexity and climate complexity [20,27,28]. Although these studies are valuable, they provide little information on TFC spatial variation in a large spatial range and fine resolution. The time period, spatial range and resolution of these studies are very limited. Some studies only focused on the spatial distribution within a small region [29], or the temporal trend of a single region [28]. At the same time, small-scale studies can only provide absolute values of the complexity of the local climate system. But in the practical evaluation, relative values of complexity among various locations are often more meaningful. Therefore, there is a necessity to obtain the spatiotemporal distribution of TFC with a longer period, larger spatial range and finer resolution. Such distribution can depict the spatial and temporal variations of TFC more accurately. However, there is no a study that provides the spatial pattern of TFC in China under a pixel resolution (like spatial resolution below 1°) to our knowledge. Our study will calculate annual temperature PE from 1979–2017 and seasonal temperature PE from 1983–2017 in China using 0.75° spatial resolution. This study can reveal the spatial variation of TFC more accurately, and compare TFC intensity among various regions.

On the other hand, it is of great significance to determine the main driving factors of the climate complexity and their influencing intension, especially for regional or large-scale climate simulation [30,31]. Detection of driving factors can help to explore the uncertainty sources in prediction results of climate change [32]. It is also helpful for researchers to analyze the influence process of various driving factors on TFC spatial variation deeply [33]. However, previous works rarely quantitatively explain the TFC spatial variation [14,27,28,29,34,35]. This is mainly because the complexity of climate system is influenced by many factors, such as atmospheric circulation, underlying surface types, solar radiation and so on. Its complexity is difficult to interpret, especially in areas with diverse topography and landforms [27,28]. For example, Hao [29] tried to explain the spatial variation of temperature PE results in Yunnan. But the influencing factors only involved topography and landform, and the analysis of these factors was only a qualitative discussion, and there was no quantitative description of their influencing intensity in his research. Clearly, driving factor analysis is the ultimate goal of spatial and temporal variation analysis of TFC. It can provide researchers insights into the mechanisms of the complexity of local climate system and climate change.

In our study, GeoDetector was introduced to interpret the spatial variation of TFC quantitatively. The method from the GeoDetector software [36] was used to quantify spatial zoning effects from co-variables. GeoDetector fills in the blank of quantitative description of spatial variation, and provides a new strategy to detect the driving factors of the spatial variation [37,38]. Various variables of spatial zoning were collected as the potential influencing factors of the TFC spatial variation, including underlying surface types, climate types, topography and terrain types, as well as regional location factor. Finally, the main influencing factors and their influencing intensities were determined.

In short, we aim to characterize the spatial pattern and temporal trend of the temperature fluctuation complexity in China from 1979–2017, and to explore the geographic causes of its spatial variation. The results can quantitatively evaluate the TFC in different regions, and give the main driving factors of the TFC spatial variation. The rest of this paper is organized as follows: The next section introduces the research materials. Section 3 describes the framework and methodology of this research. Section 4 analyzes the spatial and temporal variation of annual TFC and seasonal TFC. The results are discussed in the Section 5. The main conclusions are summarized in the last section.

## 2. Research Materials

### 2.1. Air Temperature Data

The air temperature data are derived from the 2-m air temperature dataset in the ERA-Interim reanalysis product (http://rda.ucar.edu/datasets) from the European Centre for Medium-Range Weather Forecasts (ECMWF). ERA-Interim is a global atmospheric reanalysis. This dataset provides the global grid data, with a spatial resolution of 0.75° and a temporal resolution of 6 hours (0000, 0600, 1200 and 1800 UTC). The temperature data in China (18.16–53.56° N, 73.45–135.09° E) from 1979–2017 was extracted in this research.

The accuracy of ERA-Interim reanalysis temperature datasets in China with spatial resolution larger than 0.5° has been verified by many literatures and ERA-Interim (0.75°) 2 m temperature dataset is widely used, e.g., [39,40,41,42]. For example, Gao et al. [41] and Song et al. [42] have validated the applicability of ERA-Interim temperature datasets with spatial resolution larger than 0.75° in the study of inter-annual and seasonal scale in China by comparing with air temperature records from 756 observation stations in China. Overall, the dataset with 075° spatial resolution can reflect the spatial distribution of temperatures in China with high reliability [39,42]. In addition, this study is to reveal the spatiotemporal variation of TFC. Therefore, compared with the real temperature values, the relative values of daily temperature are more meaningful in the evaluation of data applicability. Considering the uneven spatial distribution and the temporal discontinuity caused by equipment damage or station relocation of station data, this 2 m air temperature dataset was selected to analyze the spatial and temporal variation of TFC.

Annual mean temperature varies among different locations in China, ranging from −11.01–25.20 °C (Figure S1a). There are more than 50% regions in China with annual mean air temperature above 0 °C (Figure S1–S2). There is the most evident spatial variation of air temperature in winter (−25.50–20.54 °C) from Table S1. More details can be found in Support information 1.

### 2.2. Explanatory Variables of Spatial Variation of Temperature Fluctuation Complexity (TFC)

This study interprets the spatial variation of TFC from the perspectives of vegetation, climate, latitude, altitude and terrain.

**Figure 1:**
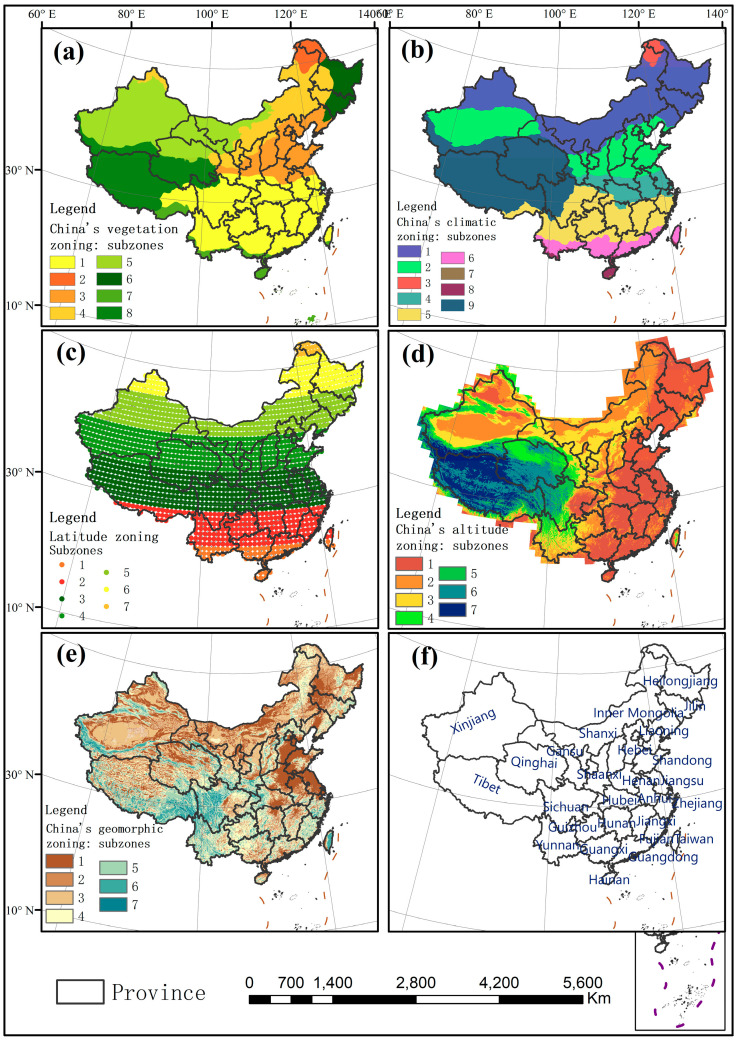
(**a**) Spatial distribution of China’s vegetation zoning with eight subzones. (1) Subtropical evergreen broad-leaved forest area, (2) Cold-temperate coniferous forest area, (3) Warm-temperate deciduous broad-leaved forest area, (4) Temperate grassland area, (5) Temperate desert area, (6) Temperate coniferous and deciduous broad-leaved mixed forest area, (7) Tropical monsoon rainforest and rainforest area and (8) Alpine vegetation area of Qinghai-Tibet Plateau. (**b**) Spatial distribution of China’s climatic zoning with nine subzones: (1) Middle Temperature Zone, (2) Warm Temperate Zone, (3) Cold Temperate Zone, (4) North Subtropical Zone, (5) Central Subtropical Zone, (6) South Subtropical Zone, (7) Middle Tropical Zone, (8) Marginal Tropical Zone and (9) Plateau Climatic Zone. (**c**) Spatial distribution of latitude zoning with seven subzones: (1) 18.75–23.75°N, (2) 24–28.5°N, (3) 29.25–34.5°N, (4) 35.25–39.75°N, (5) 40.5–45°N, (6) 45.75–51°N and (7) 51.75–53.25°N. (**d**) Spatial distribution of China’s altitude zoning with seven subzones: (1) −263–565, (2) 566–1220, (3) 1221–2045, (4) 2046–3085, (5) 3086–4050, (6) 4051–4845, (7) 4846–8535 meter. (**e**) Spatial distribution of China’s terrain zoning with seven subzones: (1) Plains, (2) Platforms, (3) Hills, (4) Small relief mountains, (5) Medium relief mountains, (6) Large relief mountains and (7) Extreme relief mountains. (**f**) The name of each province is labelled in Figure 1f.

Spatial distribution of China’s vegetation zoning is derived from the Vegetation Map (1:1 million). According to plant species, ecological types and environmental factors, eight vegetation subzones were divided: (1) Subtropical evergreen broad-leaved forest area, (2) Cold-temperate coniferous forest area, (3) Warm-temperate deciduous broad-leaved forest area, (4) Temperate grassland area, (5) Temperate desert area, (6) Temperate coniferous and deciduous broad-leaved mixed forest area, (7) Tropical monsoon rainforest and rainforest area and 8. Alpine vegetation area of Qinghai-Tibet Plateau (Figure 1a). (http://www.resdc.cn/data.aspx?DATAID=133).

Spatial distribution of China’s climatic zoning is derived from the Climatic Zoning Map of China compiled by the National Meteorological Administration of China in 1978 using climatic data from 1951–1970. According to heat, dryness, topographic characteristics and historical administrative division tradition, nine climatic subzones are divided into: (1) Middle Temperature Zone, (2) Warm Temperate Zone, (3) Cold Temperate Zone, (4) North Subtropical Zone, (5) Central Subtropical Zone, (6) South Subtropical Zone, (7) Middle Tropical Zone, (8) Marginal Tropical Zone and (9) Plateau Climatic Zone (Figure 1b). (http://www.resdc.cn/data.aspx?DATAID=243).

Latitude zoning is a self-defined zoning. In order to make the latitude range as equidistant as possible, it is divided into seven subzones: (1) 18.75–23.25° N, 2. 24–28.5° N, 3. 29.25–34.5° N, 4. 35.25–39.75° N, 5. 40.5–45° N, 6. 45.75–51° N and 7. 51.75–53.25° N (Figure 1c).

China’s altitude zoning distribution is derived from Shuttle Radar Topography Mission (SRTM) data of the US space shuttle Endeavour, with a spatial resolution of 1000m. Using the natural breakpoint method in the Geographic Information System (GIS), elevation is divided into seven subzones: (1) −263–565, 2. 566–1220, 3. 1221–2045, 4. 2046–3085, 5. 3086–4050, 6. 4051–4845, 7. 4846–8535 meter (Figure 1d). (http://www.resdc.cn/data.aspx?DATAID=123).

Spatial distribution of China’s terrain zoning is derived from the Geomorphological Atlas of the People’s Republic of China (1:1 million). Terrain subzones include (1) Plains, (2) Platforms, (3) Hills, (4) Small relief mountains, (5) Medium relief mountains, (6) Large relief mountains and (7) Extreme relief mountains (Figure 1e). (http://www.resdc.cn/data.aspx?DATAID=124).

## 3. Methods

**Figure 2:**
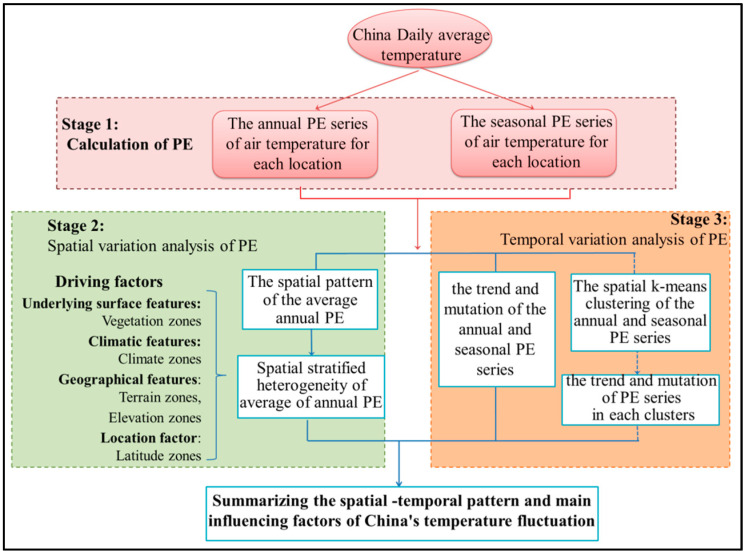
Flow chart of the research framework.

Mean daily temperate series was calculated from 2-m temperature of ERA-Interim dataset. Based on this result, the annual PE or annual TFC was calculated every year from 1979–2017 in the stage 1 (Figure 2). And the seasonal PE or seasonal TFC for each season is calculated every five years from 1983–2017 with four years overlapping.

In the next stage (Figure 2), the spatial patterns of the annual PE and seasonal PE were analyzed. Both the annual PE and seasonal PE here are the averages over the whole study period. The driving factors of TFC spatial variation were explored quantitatively by GeoDetector. Four kinds of factors were involved, including underlying surface features, climatic features, geographical features and the location.

Finally, the Mann-Kendall method was applied to analyze the trend and mutation (abrupt change) of the annual and seasonal PE series from 1979–2017 and 1983–2017 respectively in the stage 3 (Figure 2). The annual PE and seasonal PE series are the spatial average of all locations. However, the regional average may cover up the visible spatial differences of the temporal trends in various regions. Therefore, the spatial K-means clustering was also used to analyze the temporal trend of annual PE in each spatial cluster from 1979–2017 as a reference.

### 3.1. A Permutation Entropy (PE)-Based Method to Quantify TFC

PE is selected to measure the temperature fluctuation complexity. It is a natural complexity measure for time series based on comparison of neighboring values [43]. This algorithm can quantify the uncertainty of temperature changes simply and robustly. PE is calculated in MATLAB Software. The function of Permutation Entropy in MATLAB can be downloaded from (https://ww2.mathworks.cn/matlabcentral/fileexchange/37289-permutation-entropy). This function can be used directly to calculate the PE. The calculation process is as follows [43,44]:

1. Reconstruction of the phase space: For a time series of daily average temperature *x*(*i*) (*i* = 1, 2, …, *n*), we reconstructed a m-dimensional space and get: *X*(*i*) = [*x*(*i*), *x*(*i* + 1), …, *x*(*i* + (*m* − 1)*l*)]. *m* and *l* are positive integers. *l* is set to 1. *m* is crucial for the reconstruction of the phase space.
2. Recoding the reconstructed sequences: Rearrange *X*(*i*) in ascending order with [*x*(*I* + (*j*_1_ − 1)l) ≤ *x*(*I* + (*j*_2_ − 1)*l*) ≤ … ≤ *x*(*I* + (*j_m_* − 1)*l)*]. For each *X*(*i*), there is a symbolic sequence (permutation) as *A*(*g*) = [*j*_1_, *j*_2_, …, *j_m_*] (*g* = 1, 2, …, *k*), where *A* is a set of symbolic sequences for all *X*(*i*). The maximum of possible permutations is *m*!, *k* ≤ *m*!.
3. Calculation of PE: The probability of each symbol sequence is recorded as [*P*_1_, *P*_2_, …, *P_k_*]. *P_k_* is calculated as the number of occurrences of sequence *k* divided by total number of sequences. The PE of *k* symbolic sequences of time series *x*(*i*) can be defined as: $PE(m)=-\sum_{v=1}^{k}P_{v}\ln P_{v}$. When *P_v_* = 1/*m*!, *PE*(*m*) reached the maximum *ln*(*m*!). Finally *PE*(*m*) is normalized by *ln*(*m*!) and there is a more elegant form 0 ≤ *PE*(*m*) ≤ 1.

The selection of PE parameters (like, *m*) needs to make a trade-off between the validity of the time window and the length of the result sequence. Taking the research of Bandt et al. [43] as a reference, when *m* ≤ 4, PE algorithm will lose its effectiveness; if *m* is too large, it needs a long enough sequence to support, which is not practicable. At the same time, to depict the TFC trend for temporal analysis, a longer PE series need to be retained, because the time series as long as possible can improve the reliability of the temporal trend description. Therefore, *m* = 5 is selected in this study for both annual TFC and seasonal TFC calculation. Air temperature series must larger than 120 d. For seasonal PE, there is only about 90-day air temperature series for each season every year. 5 consecutive years consist the seasonal air temperature series with length about 450 d, about 4 times larger than 5!. So the stable seasonal TFC spatial pattern can be derived from the average of 35-year seasonal TFCs. But, there is only a seasonal TFCs series for 35 years and they are not independent. This is not sufficient for temporal trend analysis of TFC. Therefore, the annual PE is calculated each year independently to get independent annual TFCs for 39 years, which makes the temporal trend more valuable and reliable. The length of air temperature series for annual TFC calculation is 365 d (about 3 times larger than 5!). And the stable annual TFC spatial pattern can be derived for the average of 39-year annual TFCs.

Thus, PE here depicts uncertainty degree of the five-day temperature trend, with *m* = 5. The larger PE indicates that the temperature trend is closer to random, with a completely random situation when PE is 1. It means temperature changes in the short term are more complex and unpredictable [45]. Temperature changes may be influenced by many factors. Therefore, this also indicates that it is difficult to predict the temperature changes in this region in the next few days precisely. A small PE is opposite.

### 3.2. A GeoDetector-Based Method to Detect Driving Factors of TFC Spatial Variation

The theory of spatial stratification heterogeneity was applied here to explain the spatial variation and spatial pattern of temperature complexity. This theory proposed by Wang [46] is based on the prior spatial partitioning. Under the certain spatial partition of driving factor (X), the object of study (Y) exhibits homogeneity in the same subzones of X and heterogeneity among different subzones. In this study, Y refers to the average of the air temperature PE over the whole study period. X refers to climate, vegetation, latitude, altitude and terrain zoning.

GeoDetector is a commonly used method to quantitatively determine the driving factors of spatial heterogeneity. It is calculated in the software called GeoDetector. This software can be downloaded in (http://geodetector.org/#_Download,_with_Datasets). Its basic idea is that [47] if X has a significant influence on Y, the spatial distribution of X and Y should be similar, where X is a type variable and Y is a numerical variable. If there is strong heterogeneity of Y under the spatial zoning of X, Y displays high consistency in the same type of X and high dispersity in different types of X. Therefore the variances of Y within and between subzones can be used to judge the heterogeneity of the research object under the spatial zoning of a driving factor: $q=1-\frac{1}{N\sigma^{2}}\sum_{i=1}^{L}N_{i}\sigma_{i}^{2}$. Where *q* measures the spatial variation of Y under the spatial zoning of X (X = 1, 2, …, *L*). *L* is the number of subzones of X. σ^2^ and *N* are the variance and sample size of Y respectively. $\sigma_{i}^{2}$ and *N_i_* are the variance and sample size of Y in subzone *i* (X = *i*) respectively.

*q* is from 0–1. When $\sigma_{i}^{2}$ approaches zero, the homogeneity within subzones is the strongest and *q* approaches 1. When there is no significant difference between $\sigma_{i}^{2}$ and $\sigma_{i}^{2}$, the partition variable X has no explanatory power for Y and *q* approaches 0.

In addition to measure the single factor influence, this method can also quantify the effect of the interaction between the two factors on the spatial variation of Y. The estimation of *q* of two-factor interaction is the same as that of single-factor *q*, except that the spatial zoning of X is formed by two-factor intersect (X = X1 ∩ X2) in two-factor interaction estimation. Considering the complex interaction between various factors and non-linear superposition of their effect, it will be more reasonable to analyze the spatial variation of TFC under the interaction of two factors through their intersection.

### 3.3. Mann-Kendall Method to Investigate TFC Temporal Variation

The Mann-Kendall method is a non-parametric statistical test method [48]. Compared with the parametric statistics, it has advantages that the time series does not need to obey certain distribution rules and the result is merely disturbed by outliers [49]. Mann-Kendall method is conducted in MATLAB Software by function called the sequential Mann-Kendall test. This function can be downloaded from (https://ww2.mathworks.cn/matlabcentral/fileexchange/63708-sequential-mann-kendall-test). It can be used directly to detect find the potential trend turning point in long term datasets. The specific calculation process is as follows [50]:

1. Given PE series {*PE_i_*} i = 1, 2, …, n, where n is 39 for annual PE series and n is 35 for seasonal PE series, *S_k_* is the number of *PE_i_* exceeding *PE_j_* (1 ≤ *j* ≤ *i*).
  (1) $$S_{K}=\sum_{i=1}^{k}r_{i}\;(i=1,\;2,\;\ldots ,\;n);\;r_{i}=\left\{ \begin{array}{l} 1\;PE_{i}>PE_{j}\\ 0\;PE_{i}\leq PE_{j} \end{array}\right.\;(j=1,\;2,\;\ldots ,\;i)$$
2. {*UF*} is calculated to depict the trend from *PE*_1_ to *PE_k_*. Under the assumption of random and independence of time series, *UF_k_* (*k* = 1, 2, …, *n*) quickly converges to the standard normal distribution as *n* increases (*n* > 10),
  (2) $$UF_{k}=\frac{S_{k}-E(S_{k})}{\sqrt{Var(S_{k})}},\;E(S_{k})=\frac{n(n-1)}{4},\;Var(S_{k})=\frac{n(n-1)(2n+5)}{72}$$
3. {*UB*} is calculated to depict the trend from *PE_k_* to *PE_n_*. Reverse the sequence {*PE_i_*} and repeat the step2 to get *UF’_k_*.
  *UB_k_* = −*UF’_k_*., where *UB*_1_ = 0 and *k* = *n*, *n* − *1*, …, 1.

There is an upward trend from *PE*_1_ to *PE_k_* when *UF_k_* > 0. *UF_k_* < 0 indicates a decline. Based on the hypothesis test (null hypothesis is {*PE_i_*} is completely random), null hypothesis is rejected if |*UF_k_*| > *Z*_0.05/2_ there is significant trend of the PE sequence. |*UF_k_*| < *Z*_0.05/2_ is the opposite. From the normal distribution table, *Z*_0.05/2_ is 1.96. If the two curves of the {*UF*} and {*UB*} have an intersection between the significant lines, there is the mutation of {PE_i_} in the year of the intersection point of {*UF*} and {*UB*}. That means the intersection point is the beginning of the abrupt change of {PE_i_}. 

## 4. Results and Analysis

The PE of air temperature quantifies the TFC. Therefore it can be used to measure the predictability of the temperature trend for a certain region. In this section, the spatial variation and its driving factors for the annual TFC and seasonal TFC will be illustrated in Section 4.1 and Section 4.2 respectively. Meanwhile, the temporal variation of the annual and seasonal TFC will be analyzed in Section 4.3.

### 4.1. Spatial Variation of Annual TFC and Its Driving Factors Analysis

**Figure 3:**
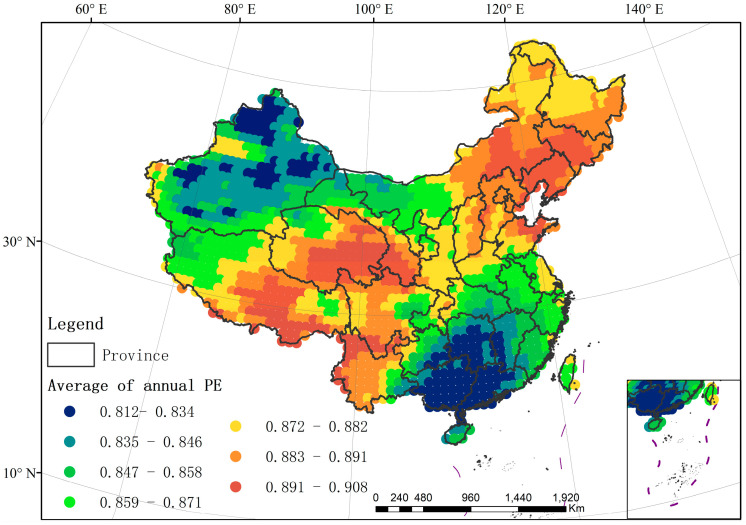
Spatial distribution of the annual temperature fluctuation complexity (TFC) (the average of annual permutation entropy (PE) from 1979–2017).

The annual TFC in China shows the obvious spatial variation, ranging from 0.812–0.903 (Figure 3). The annual TFC is lower in the Northwest (Xinjiang Basin) and Southeast (Hunan, Jiangxi, Guangxi and Guangdong) of China. Their PE is basically below 0.85. It is indicated that the temperature series in these regions are more regular. Temperature changes of five days are less complex. In other words, the specific trend is much more frequent than the others in the regions with low TFC or low PE. Taking results in Table S2 as a reference, trend number 109 is much more frequent than other trends. Therefore it is easier to accurately predict the temperature changes in the next five days in these regions. 

The annual TFC is higher in northeastern China (Jilin, Liaoning, northeastern Inner Mongolia), and in southwestern China (Qinghai, Tibet, Yunnan and Sichuan), which is basically higher than 0.85. It means that the temperature series are more random in these regions. There are more possible trend states of five-day temperature change, with high complexity of temperature changes. Therefore, it is difficult to accurately predict the temperature changes in the next five days in these regions. In addition, it should be noted that the similarity of PE values in the two regions can only reflect that the temperature trends in these two regions have similar uncertainties, rather than identical trends or identical actual temperature states. For example, PE is similar in Xinjiang Basin and southeastern China, and the TFC in these areas is similar, but their actual probability distributions of temperature trends are different (details can be found in Table S2).

**Figure 4:**
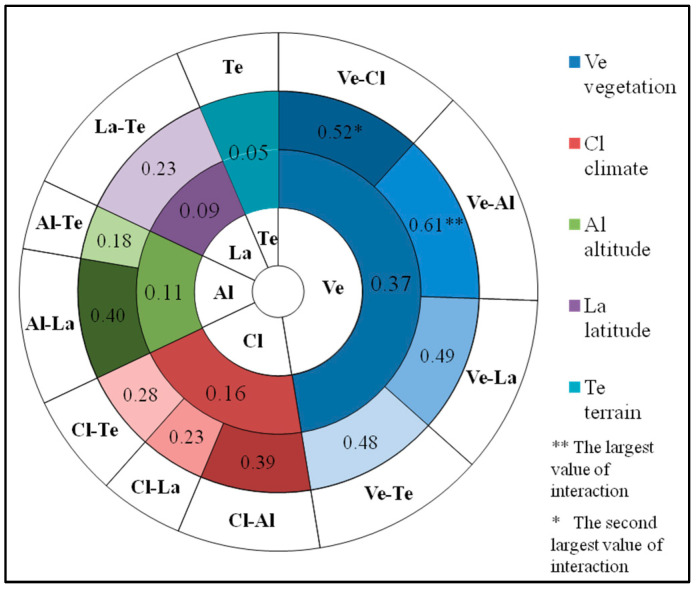
Driving factor explaining ability of spatial variation annual TFC. The inner ring corresponds to the explaining ability of each single factor, and the outer ring corresponds to the explaining ability of the interaction between two factors. A–B represents the interaction between A and B.

The results of GeoDetector quantify the influence intensity of single factor (climate, vegetation, terrain, altitude and latitude) and two-factor interaction on annual TFC (Figure 4). Vegetation, climate and elevation are the main driving factors for spatial variation of annual TFC. Generally, vegetation is the most powerful driving factor for spatial variation of annual TFC, which can explain 37% of its spatial variation. The explaining ability of climate type is the second, which can explain 16%. Altitude and latitude can explain 11% and 9% of annual TFC spatial variation respectively. Terrain shows the weakest influence on spatial variation of annual TFC, with only 5%. It can be seen that the influence of vegetation and climate is much higher than that of other factors. From the perspective of two-factor interaction, 52% spatial variation of annual TFC can be explained by the vegetation-climate interaction. Their interaction influence exceeds the maximum of their single factor influence, but is less than the linear summation of their single factor influence, with two-factor enhancement. The vegetation-elevation interaction achieves the best interpretation for spatial variation of annual TFC (61%). Their interaction exceeds the sum of their single factor influence (37% + 11% = 48%), which shows the two-factor non-linear enhancement.

The annual TFC is various in different subzones of each driving factor, which leads to the spatial variation of annual TFC. The annual TFC intensity of each sub-region for all factors is described in Tables S3–S4. In terms of vegetation zoning, the predictability of temperature changes in temperate desert region is the highest, with the average annual-PE about 0.85. There is the second highest predictability in the subtropical evergreen broad-leaved forest region and the tropical monsoon rainforest-rainforest region, with annual PE ranging from 0.86–0.87. The intensity of annual TFC fluctuates basically around 0.88 in the temperate coniferous forest region, the alpine vegetation region at Qinghai-Tibet Plateau, the temperate grassland region and the warm temperate deciduous broad-leaved forest region. The uncertainty of temperature changes is the highest in temperate coniferous and deciduous broad-leaved mixed forest region, with annual-PE about 0.89. In climate zoning, there is the lowest annual TFC in the middle tropical zone and south subtropical zone, averaging about 0.84–0.85. The second largest annual TFC can be found in marginal tropical zone, middle subtropical zone, north subtropical zone and warm temperate zone, and annual-PE is 0.86 basically. The intensity of annual TFC in middle temperate zone is around 0.87. The uncertainty of temperature changes is the highest in plateau climate zone and cold temperate zone, with annual-PE about 0.88. In altitude zoning, the uncertainty of temperature changes increases with altitudes generally. The intensity of annual TFC is the lowest in Altitude Level 2, averaging about 0.86. The annual TFC is the strongest in Altitude Level 5–6 about 0.88.

The vegetation, climate and altitude are the critical driving factors for spatial variation of annual TFC. The various combinations of their sub-regions cause the different interaction processes at different regions. There are also similar or opposite annual TFC intensities in the regions with different geographic environments.

Both the annual TFC in Northwest and Southeast China are weaker than the other regions. It indicates that the short-term temperature changes in Northwest and Southeast China are less complex and more regular than that in the other regions. The temperature changes in Northwest and Southeast China may be not easy to be effected by outside factors. Therefore, it is not difficult to predict the temperature changes in these regions in the next few days precisely. However, the geographic environments of these two regions (Northwest and Southeast China) are different, and the reasons for their weak complexity are different from Figure 1 and Figure 3. For Xinjiang Basin in Northwest China, the low uncertainty of temperature changes is mainly related to underlying surface characteristics and altitude. The vegetation type of this area is temperate desert, and this basin area has a low altitude, basically lower than 1220 m (Altitude Level 1–2). In the middle of Tarim Basin, the uncertainty of temperature changes is the lowest with PE ranging from 0.812–0.834 and elevation of −263–565 m (Altitude Level 1). In Junggar Basin, the annual TFC is a little stronger ranging from 0.835–0.845, with the elevation of 566–1220 m (Altitude Level 2). In the Tianshan Mountains of Xinjiang, its temperature changes are complex, with high elevation (Altitude Level 4–6). The climate types are mainly the mid-temperate climate and warm-temperate climate in Xinjiang. It can be seen that climate has little influence on annual TFC in this region. For Southeast China, its weak annual TFC is mainly affected by the altitude, vegetation and climate. The overall altitude in this area is relatively low, about −263–565 m (Altitude Level 1). In Guangxi and Guangdong, the intensity of annual TFC is the lowest, ranging from 0.812–0.834. Climate types are mainly south subtropical climate in the central and southern part of this region, and the middle subtropical climate in the north. The vegetation types are subtropical evergreen broad-leaved forest, tropical monsoon rainforest and rainforest region. In Hunan and Jiangxi, the TFC intensity is lower than 0.845. The vegetation type is subtropical evergreen broad-leaved forest. The climate type is the middle subtropical climate.

On the other hand, both the annual TFC in Northeast and Southwest China are stronger than the other regions. However, the geographic environments of these two regions are different, and three dominant driving factors present different influence processes in these two regions from Figure 1 and Figure 3. For the northeastern China, the strong uncertainty of temperature changes is mainly affected by the vegetation and climate factors. In Jilin, Liaoning and northeastern Inner Mongolia, the vegetation types are temperate grassland, temperate coniferous-deciduous broad-leaved mixed forest and warm temperate deciduous broad-leaved forest. The climate type is the middle temperate climate. In Qinghai and Tibet, the strong annual TFC is mainly affected by altitude, vegetation and climate. Most of the areas are above 4000 m (Altitude Level 6–7) in this region. Climate type is the plateau climate and the vegetation type is the alpine vegetation. In Yunnan and Western Sichuan, the strong annual TFC is related to altitude and terrain. The region has a high elevation of more than 2000 m (Altitude Level 3–7). Its terrain types are small-extreme relief mountains (Terrain Type 4–7).

### 4.2. Spatial Variation of Seasonal TFC and Its Driving Factors Analysis

We selected the months from March to May, June to August, September to November, and December to February as spring, summer, autumn and winter, respectively.

**Figure 5:**
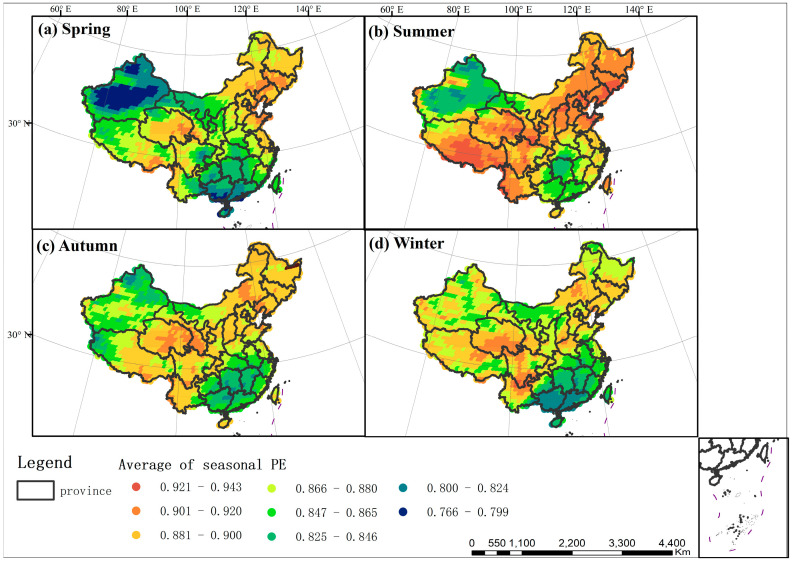
Spatial distribution of seasonal fluctuation complexity of air temperature (the average of seasonal PE from 1983–2017). (**a**) Spatial distribution of spring TFC in China. (**b**) Spatial distribution of summer TFC in China. (**c**) Spatial distribution of autumn TFC in China. (**d**) Spatial distribution of winter TFC in China.

The intensity of seasonal TFC is different in four seasons (Figure 5). Overall, there is the strongest TFC in summer, with PE ranging from 0.811–0.943. The uncertainties of temperature changes in autumn and winter rank second and third respectively. The TFC in spring is the weakest (0.766–0.914). It is indicated that five-day temperature changes are less complex and easier to accurately predict in spring. 

Meanwhile, the seasonal TFC intensity in China also shows obvious spatial variation, ranging from 0.766–0.943 (Figure 5). In spring, the weak TFC is concentrated in Xinjiang Basin, southern of Guangxi and Guangdong, with seasonal PE from 0.766–0.799; the strong TFC is concentrated in northeastern China including Jilin, Liaoning and Eastern Qinghai, and seasonal PE ranges from 0.901–0.920 in Figure 5a. In summer, the uncertainty of temperature changes is low in Xinjiang Basin, Hunan, Guangxi and Guangdong, with seasonal PE from 0.825–0.865; the uncertainty is high in southwest China (Jilin, Liaoning, Hebei, Shanxi and Shandong), and seasonal PE changes from 0.901–0.943 in Figure 5b. In autumn, temperature changes are more regular in the northern and southern of Xinjiang Basin and the southeast area, with seasonal PE from 0.825–0.865; temperature changes are more complex in the northeast area, Qinghai and southern Gansu, with seasonal PE from 0.881–0.920 in Figure 5c. In winter, the TFC is weak in Guangxi and Guangdong, with seasonal PE from 0.800–0.824, and strong in Northeast Inner Mongolia, Jilin, Liaoning, Qinghai and Sichuan, with seasonal PE from 0.881–0.920 in Figure 5d.

**Figure 6:**
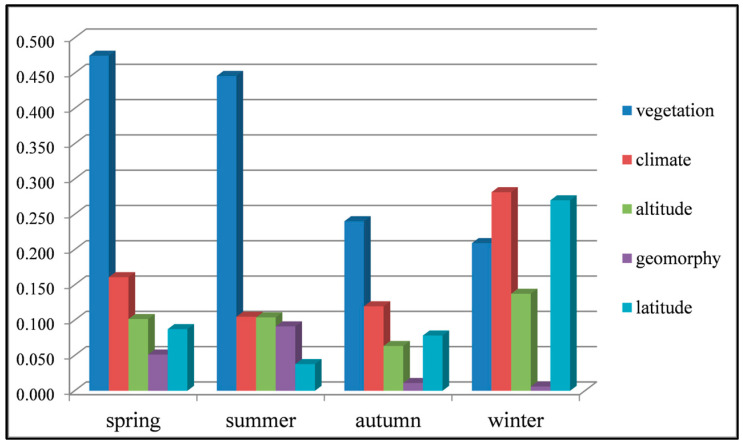
Single factor explaining ability on spatial variation of seasonal TFC.

**Figure 7:**
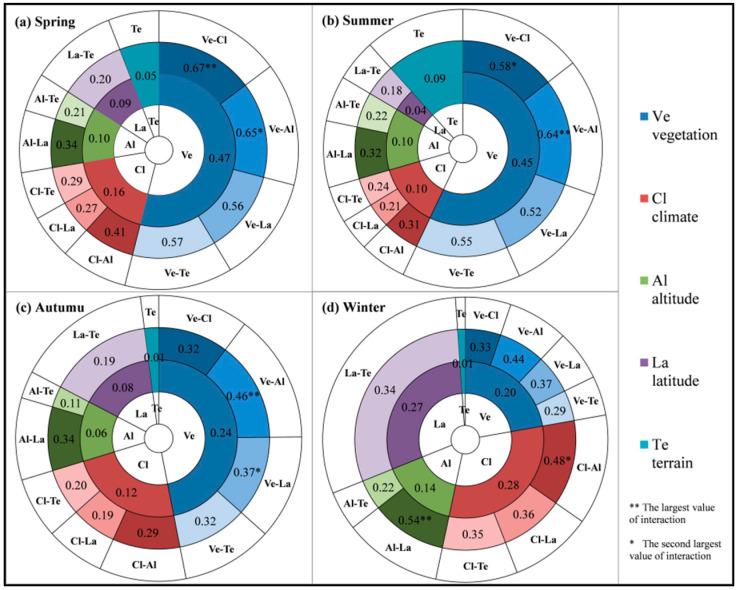
Driving factor explaining ability on spatial variation of seasonal TFC. The inner rings correspond to the explaining ability of each single factor, and the outer rings correspond to the explaining ability of the interaction between two factors. A–B represents the interaction between A and B. (**a**) Driving factor explaining ability on spatial variation of spring TFC. (**b**) Driving factor explaining ability on spatial variation of summer TFC. (**c**) Driving factor explaining ability on spatial variation of autumn TFC. (**d**) Driving factor explaining ability on spatial variation of winter TFC.

The single factor and the interaction between two factors display different influencing effects on the TFC in different seasons (Figure 6 and Figure 7). The main factors of spatial variation of seasonal TFC are not identical in four seasons. From summer to winter, the influence of vegetation and terrain on seasonal TFC is gradually weakening, whereas the influence of climate and latitude on seasonal TFC is strengthening. By spring, the influence of these factors has changed dramatically, and their influencing intensities on TFC are similar to those on summer TFC. There is similar influencing effect of elevation in different seasons, while with the strongest influence in winter.

The explanatory effects of all factors on TFC spatial variation in the four seasons were described respectively. In spring, vegetation is the dominant driving factor for TFC spatial variation and can explain 47% of its spatial variation. The explaining ability of climate zoning is second, which can explain 16%. Altitude and latitude can interpret 10% and 9% of spatial variation of spring TFC respectively. There is the weakest influence on spring TFC in terrain zoning with 5%. From the perspective of interaction in Figure 7a, there is the strongest influence of vegetation-climate and vegetation-elevation interaction on spring TFC. They can explain 67% and 65% of its spatial variation respectively, with two-factor non-linear enhancement. In summer, the main driving factor of TFC spatial variation is vegetation type, which can explain 45%. The explaining ability of climate and altitude comes to the next, with 10%. Terrain can explain 9% of TFC spatial variation in summer. Latitude can only explain 4%. In addition, from Figure 7b, there is the best interpretation effect of the interaction between vegetation and altitude on summer TFC. Their interaction can explain 64% of spatial variation in summer. In autumn, vegetation type is the critical driving factor for TFC spatial variation, with explanation degree of 24%. The explaining ability climate type is the second (12%). Latitude and altitude can interpret 8% and 6% of TFC spatial variation in autumn. Terrain can only explain 1%. From the interaction in Figure 7c, the best explanatory effect can be found in the interaction between vegetation and altitude (46%), which shows the non-linearity enhancement. The explaining ability of altitude-latitude interaction is second, with 34%. In winter, climate and latitude become the critical factors of TFC spatial variation. They can explain 28% and 27% of winter TFC spatial variation respectively. The explanatory power of vegetation zoning is the second, with 20%. Altitude can interpret 14%. Terrain can only explain 1%. From the interaction of two factors in Figure 7d, the interpretation effect of altitude-latitude interaction is the best, which can explain 54%. The interaction between altitude and climate takes the second place, with 48%. It is interesting that the vegetation has weaker influence in winter markedly than that in other seasons.

The seasonal TFC is various in different subzones of each driving factor, which leads to the spatial variation of seasonal TFC in four seasons (from Figure 1 and Figure 5). The seasonal TFC intensity of each sub-region for all factors is described in Table S5. In Xinjiang Basin, there is low altitude and temperate desert vegetation type. The influence of vegetation-elevation interaction is the most powerful in spring, summer and autumn. Therefore, low uncertainty of temperature changes is mainly manifested in these three seasons. In Southeast China (Guangxi, Guangdong, Hunan, Jiangxi), the TFC of all four seasons is relatively weak. This main reason is its low altitude, low latitude. And climate types in this region are the middle-subtropical, south-subtropical, middle-tropical and marginal tropical climate. The vegetation types are the subtropical evergreen broad-leaved forest, tropical monsoon rainforests and rainforests. In addition, there is the greatest influence of the altitude-latitude interaction in winter. Therefore, the weak TFC of the southeast is more apparent in winter. In Northeast China (Jilin, Liaoning, Northeast Inner Mongolia), there is relatively high uncertainty of temperature changes in four seasons. The reason for this is its high latitude and mid-temperate climate. Its vegetation types are grassland, coniferous forest and deciduous broad-leaved forest in this region. In Qinghai and Tibet, the TFC is relatively strong in four seasons, because of their high altitude (above 4000 m), complex terrain, plateau climate and alpine vegetation. Meanwhile, terrain displays the strongest influence in summer. Terrain in southern Tibet is more complex than that in southern Qinghai and other regions, with mainly large relief mountains and extreme relief mountains. Therefore, the strong TFC in Tibet is more apparent in summer.

### 4.3. Temporal Variation of Annual TFC and Seasonal TFC

**Figure 8:**
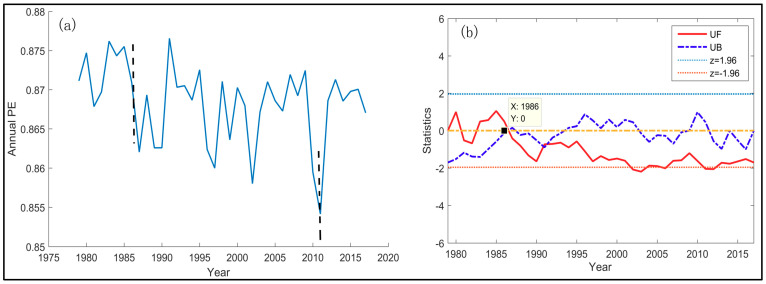
(**a**) Annual mean series of annual temperature fluctuation complexity over 1979–2017. (**b**) Temporal trend and mutation of annual temperature fluctuation complexity over 1979–2017. The series of annual PE here is the average of annual PE series at all locations in China, and annual PE series at each location is calculated in Section 3.1. Two black dash lines in Figure 8a correspond to two special years, the mutation starting year 1986 and the lowest PE year 2011 respectively. The black point in Figure 8b marks the intersection of *UF_k_* and *UB_k_* between the significant lines, which is noted as the mutation starting year.

There is distinct temporal variation of annual TFC. The average annual PE declined obviously over 1979–2017. This means that the uncertainty of 5-day temperature changes in China showed a downward trend from 1979–2017 generally. Moreover, the annual TFC reached the lowest value of the whole study period in 2011 (Figure 8a). From Figure 8b, two curves of the *UF_k_* and *UB_k_* had one distinct intersection between the significant lines (*Z*_0.05/2_ = +1.96) in 1986. It is indicated that there is the mutation of TFC intensity in 1986. In other words, the abrupt change of annual TFC started in the year of 1986. Since 1986, *UF_k_* had been below 0. And *UF* was below −1.96 in 2011. This indicates that the annual TFC decreased substantially from 1979–2011. Meanwhile, the *UB* in 2011 was over 0, but less than 1.96. It means that there was an insignificant increase of annual TFC from 2011–2017.

**Figure 9:**
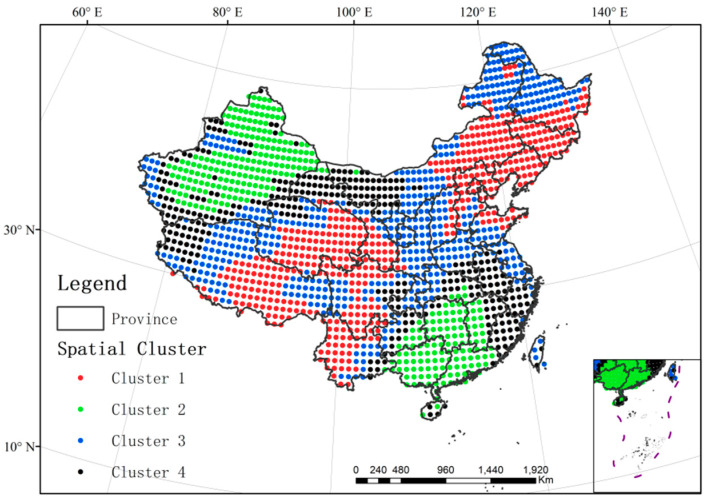
Spatial distribution of four annual TFC clusters in China.

**Figure 10:**
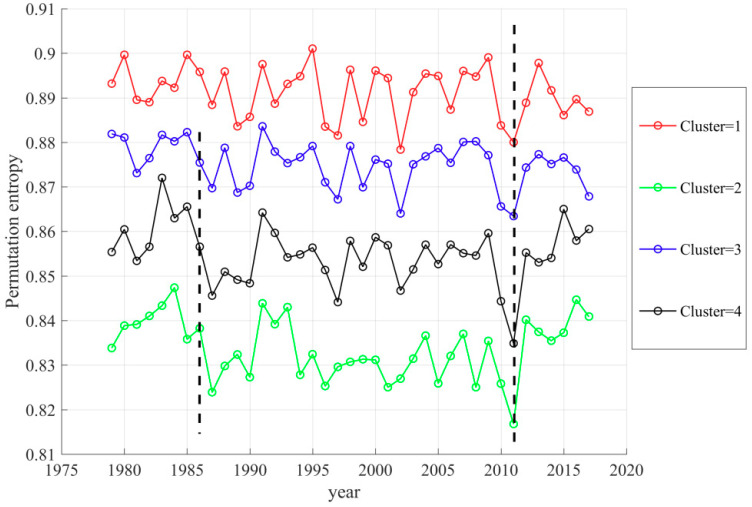
Spatial average time series of four annual TFC clusters over 1979–2017. The black dash line in 1986 marked the mutation of TFC intensity. The black dash line in 2011 marked the lowest TFC intensity.

The series of annual TFC in Figure 8a is the average of PE series at all locations. Nevertheless, such regional average may cover up the dramatic spatial variation of temporal trends in different regions. Therefore, the spatial K-means clustering was used to analyze the annual TFC trend of each spatial cluster from 1979–2017. In K-means clustering, four is found to be the optimal cluster number (Figure S3) and the spatial distribution of these four clusters is described in Figure 9. For each sub-region, we calculated the average PE within the cluster at each year and obtain the time series over 39 years for four sub-regions (Figure 10). The black dash line in 1986 marked the mutation of TFC intensity. The black dash line in 2011 marked the lowest TFC intensity. The results prove that the trend of annual TFC in all clusters is similar to that of average annual TFC and they all reached the weakest TFC in 2011 (Figure 10). There had been the strongest TFC in Cluster 1 and the weakest TFC in Cluster 2 from 1979–2017. Except for Cluster 1, the mutation of TFC series started in 1986 in the other three clusters (Figure S4). *UF_k_* kept on less than −1.96 after 1986 in the Cluster 2–4 and their TFC showed the downward trend. The mutation process of Cluster 1 is quite complex and its trend is not clear. *UF* and *UB* have more than one intersection between the significant lines (Figure S4a). There was the most evident downward trend of annual TFC from 1979–2017 in Cluster 2, with the weakest TFC. *UF_k_* in 2000–2011 had been lower than −1.96 and the annual TFC declined strikingly in Cluster 2 from 1979–2011. In general, the trend and mutation results of regional average TFC are representative.

**Figure 11:**
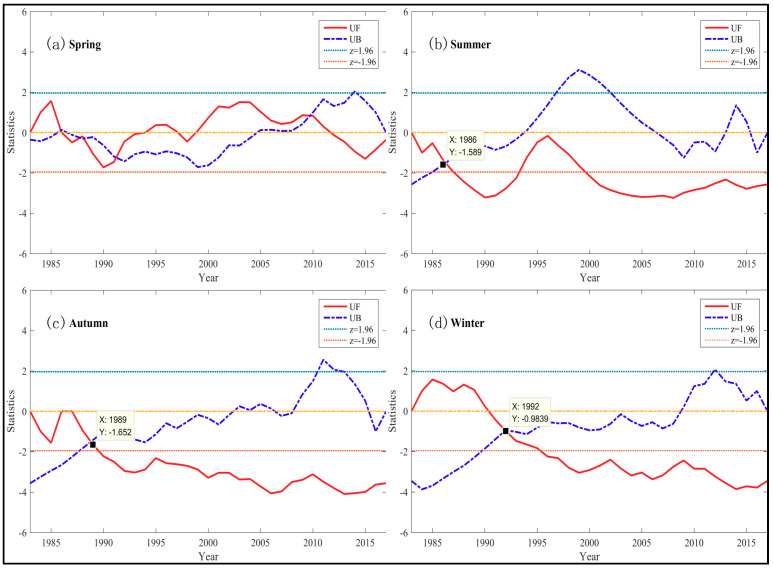
Temporal trend and mutation of seasonal temperature fluctuation complexity over 1983–2017. The series of seasonal PE here is the average of seasonal PE series at all locations in China. The seasonal PE series at each location is calculated in Section 3.1. The points marked in Figure 9b–d are the intersection of *UF_k_* and *UB_k_* between the significant lines, which are noted as the mutation starting years. (**a**) Temporal trend and mutation of spring TFC over 1983–2017. (**b**) Temporal trend and mutation of summer TFC over 1983–2017. (**c**) Temporal trend and mutation of autumn TFC over 1983–2017. (**d**) Temporal trend and mutation of winter TFC over 1983–2017.

There is distinct temporal variation of seasonal TFC. In addition to spring, the other seasons showed a downward trend generally from 1983–2017. The second half of the *UF* sequence was lower than the confidence line of −1.96 (Figure 11b–d) in the other seasons. In spring, mutation characteristics of TFC series are complex and there is on significant trend (Figure S5a). There is more than one intersection between the significant lines (Figure 11a). In summer, autumn and winter, the seasonal TFC reached the lowest level in 2001, 2011 and 2012 respectively (Figure S5b–d), where their *UB* are over 1.96 and their *UF* are below −1.96. The weakest spring TFC occurred in 1990 and 2014 (Figure S5a). 

However the series of seasonal TFC in Figure S5 is also the average of seasonal PE series at all locations. As with the clustering analysis for annual TFC, the spatial K-means clustering was also used to analyze the seasonal TFC trend of each spatial cluster from 1983–2017 in case that such regional average may cover up the obvious spatial variation of temporal trends in different regions. The detailed information about the seasonal clustering results can be found in Support information 5.

For each season, the results prove that the trend of seasonal TFC in all clusters is similar to that of average seasonal TFC. And the time of the lowest seasonal TFC for each cluster is identical with that for regional average series (Figures S5–S8). In general, the trend analysis of regional average of the seasonal TFC is representative.

The trend and mutation characteristics of the seasonal TFC in China are various in different seasons. In spring, there is no significant change trend of TFC and its TFC fluctuates around 0.858 (Figure S5a). The trend and mutation characteristics of TFC in summer, autumn and winter are similar to those of annual TFC series. *UF_k_* and *UB_k_* have an intersection between the significant lines in these three seasons. There is a significant abrupt change of TFC series and their starting year of the abrupt change was basically 1986–1992. In summer, the abrupt change of summer TFC started in 1986 (Figure 11b). There was a downward trend from 1983–2001 (with summer TFC from 0.892–0.877). Then summer TFC rose to 0.887 from 2001–2017 (Figure S5b). In autumn, the abrupt change of TFC series started in 1989 (Figure 11c). Autumn TFC decreased markedly from 0.881–0.867 in 1983–2011. Then it ascended to 0.873 after 2011 (Figure S5c). In winter, the abrupt change of TFC series started in 1992 (Figure 11d). Winter TFC experienced an apparent decline in 1983–2012, dropping from 0.873–0.867, and increased to 0.875 after 2012 (Figure S5d). 

## 5. Discussion

The spatial variation of TFC has attracted a lot of attention these days e.g., [27,51,52]. The results of this study show that the spatial variation is mainly manifested in the five regions and the spatial pattern revealed in this research can be verified in the research of Li et al. [12]. The TFC spatial pattern and reasons as follows: (1) the TFC in the Northwest (Xinjiang Basin) is relatively weak, especially in spring, summer and autumn. The short-term temperature trend in this region is predictable. This could be attributed to its low altitude and temperate desert. (2) The TFC in Hunan, Jiangxi, Guangxi and Guangdong of the southeast is weak in four seasons, and is more evident in winter. The short-term trend of temperature in this region is also easy to predict accurately. This is mainly due to its low altitude and low latitude. Climate types are the mid-subtropical, sub-subtropical, and mid-tropical climate. Vegetation types are the evergreen broad-leaved forest and rainforest. (3) The TFC in Jilin, Liaoning and northeastern Inner Mongolia is quite strong in the four seasons. The predictability of the short-term temperature trend in this region is poor. Apart from its high latitude and mid-temperate climate, the vegetation types are grassland, coniferous forest and deciduous broad-leaved forest. (4) The TFC in Qinghai and Tibet of southwestern China is strong in four seasons. The short-term temperature changes in Qinghai and Tibet are also less predictable. This is mainly because of its high altitude (above 4000 m), plateau climate, and alpine vegetation of the Qinghai-Tibet Plateau. At the same time, the southern Tibet is characterized by complex terrain and the strongest influence of terrain in summer. Therefore, the strong TFC of Tibet is more apparent in summer. (5) The other central regions have moderate TFC and moderate predictability of short-term temperature changes.

As the researchers discussed in previous literature, TFC may be affected by many factors, such as underlying surface, climate, topography and geomorphology, spatial location [53,54,55]. However, from the results illustrated in the previous section, there are spatial variations in the influencing intensity and process of these factors on TFC. On one hand, the TFC in the two regions is similar, but the natural environment their background may be very different. The different influencing processes of the driving factors have produced similar temperature fluctuation complexity [56]. On the other hand, the different combination of subzones of factors can also produce different TFC intensity [54].

From the view of vegetation zoning, its influence intensity on TFC displays the influence on spring TFC > that on summer TFC > that on annual TFC > that on autumn TFC > that on winter TFC. The weakest influence of vegetation in winter is mainly due to the vegetation withering and snow cover in winter. Therefore, there is little difference on vegetation status of each region. The regional difference of vegetation is smaller in winter than that in spring and summer [56]. Different vegetation cover types correspond to the properties of land underlying surface (albedo, roughness, etc.), which affect the global land-atmosphere energy cycling process [57]. Therefore, the TFC in different vegetation types is different. TFC in the temperate desert region and subtropical evergreen broad-leaved forest region is the weakest, basically less than 0.86. Short-term temperature changes in temperate grassland area, deciduous broad-leaved forest area and coniferous forest area is the most complex, with PE exceeding 0.88.

From the climate zoning, the TFC in the middle and south tropical zones is the weakest, averaging about 0.84–0.85. TFC in marginal tropical, middle subtropical, north subtropical and warm temperate zones is basically 0.86. The TFC in middle temperate zone is around 0.87. The strongest TFC is in plateau climate zone and cold temperate zone, with average PE of about 0.88. Short-term temperature changes are more complex in temperate zone than those in subtropical zone. This discovery was also demonstrated in the research of Wang H. et al. [58].

Latitude zoning reflects differences in local solar radiation. Generally speaking, the lower the latitude, the greater the solar altitude angle and the stronger the solar radiation; conversely, the higher the latitude, the weaker the solar radiation [59]. With the increase of latitude, the TFC shows an overall upward trend. In latitudinal belt 18.75–28.8° N, there is lower uncertainty of 5-day temperature changes, with seasonal PE less than 0.86. In latitudinal belt 45.75–53.25° N, the seasonal PE is higher than 0.87, displaying more complex climate change. Latitude has the strongest explanatory power in winter, and this result was confirmed by He et al. [54].

Altitude and terrain depict geographical features. Strong TFC always occurs in areas with high altitude and complex terrain character, which makes it difficult to estimate and simulate temperature changes accurately in these areas [11,60]. In the regions with altitude less than 1220 m (Altitude Level 1–2), the TFC is the weakest, averaging about 0.87. In the regions with altitude 3086–4845 m (Altitude Level 5–6), there is the strongest uncertainty of temperature changes, with PE over 0.88. Although there is little difference of TFC among various terrain types, the TFC increases with the terrain complexity generally.

Our study assessed the complexity of temperature fluctuations over the past 30 years. However, we do not predict and analyze the possible changes in TFC spatial pattern in the future. This requires the further analysis. The vegetation and the climate play an important role in TFC. Against the background of dramatic global change, there is considerable change on vegetation growth process and climate state [61,62]. For example, the current temperature increase in winter may conduce to soil thawing and mineral dissolution in the alpine region. This will improve water and heat conditions, advance vegetation germination and promote vegetation growth [63]. This may cause the changes of annual TFC spatial pattern. This may also strengthen the influencing effect of vegetation on winter TFC and make it become one of the most important factors in winter, thus affecting the spatial pattern of winter TFC. 

In addition to the factors involved in this study, other factors may also have an impact on the spatial variation of TFC. For example, the spatial variation of TFC is apparent in the direction of northeast-southwest. This pattern suggests that TFC spatial variation may be driven by the monsoon. The monsoon is an important factor affecting the climate, especially in the southeast of China in summer [64,65]. To accurately describe the TFC spatial variation driven by the monsoon, the precise zoning of the monsoon area needs to be collected to reveal the spatial distribution of different monsoon types and their influence intensity. On the other hand, this research does not discuss the effect of urban land cover on TFC. With development of economic and the civil society, cities has been identified as one of the landmarks for climate change [66]. Human-driven impacts may have strong explanatory power on TFC spatial variation. This deserves further study.

The temporal variation of TFC from 1979–2017 is also analyzed in this research. There are some changes over these 39 years. The average annual PE declined considerably over 1979–2017. And the annual TFC in Cluster 2 and Cluster 4 descended more quickly than the other regions. This can be influenced by many factors, such as the vegetation changes [53], the regional climate changes [64] and even the human-driven factor [67]. For example, the TFC trend in the northeast of the Cluster 2 and Cluster 4 may be mainly influenced the intensity of desertification. In the research of Tao et al. [66], desertification developed generally in northeast China from 1986 to 2000 because climate conditions became more and more unfavorable for vegetation activities. However the desertification process was transforming from development to stability from 1986 to 2000 due to the positive role of human activities like desertification control and ecological environment construction work [67]. These findings are consistent with the temporal trend of TFC in Cluster 2 and Cluster 4. The TFC in Cluster 2 and Cluster 4 decreased before 2000 and the decline slowed around 2000. Besides the increase of TFC in northeast of Cluster 2 and Cluster 4 after 2010 may be related to effective desertification management in recent years.

The influencing intensity of terrain can also be affected by the spatial resolution of air temperature. In this research, we use the ERA-Interim 2 m air temperature dataset with 0.75 spatial resolution. The analysis of this dataset in ERA-Interim is performed in separate steps following the upper-air atmospheric 4D-Var analysis. These components of the data assimilation system use relatively simple data interpolation schemes [68]. This makes ERA-Interim with finer spatial resolution like 0.25° perhaps unable to describe the temperature spatial changes precisely and objectively. In the future, researchers can collect the air temperature with finer spatial resolution and higher accuracy to improve the analysis of terrain influence on TFC spatial variation.

## 6. Conclusions

In this study, we propose a framework to analyze TFC. First, we quantify the annual-TFC from 1979–2017 and seasonal-TFC from 1983–2017 in different regions of China by PE. Then their spatial variations are revealed and main driving factors of TFC spatial variations are detected by GeoDetector. Finally, their temporal trend is described by Mann-Kendall method. The main conclusions are as follows:

1. This framework to explore the driving factors on TFC is feasible and effective.

2. TFC in China presents obvious time variation. The uncertainty of temperature changes showed a downward trend from 1979–2017 in China. The uncertainty reached the lowest value in 2011. For a certain season, the trend characteristics of TFC in summer, autumn and winter is similar to that of annual TFC. There is no significant trend for spring TFC.

3. TFC also shows clear spatial variation in China. Annual TFC intensity changes from 0.812–0.903. The seasonal TFC varies from 0.766–0.943 at different locations. Generally summer TFC is the strongest and the spring TFC is the weakest. The spatial variation of the TFC is mainly reflected in the five regions. There is low TFC in the northwest and southeast. There is high uncertainty of temperature change in northeastern and southwestern regions. TFC in the central region is moderate

4. The explanatory powers of each potential driving factor on TFC are various. The interaction between two factors has more powerful impact than the single factor. The explanatory ability of each driving factor is lower than 50% of TFC spatial variation. Vegetation is the main driving factor of TFC spatial variation, followed by climate and altitude. Latitude and terrain have the lowest impact. The influencing intensity of each single factor varies in four seasons. From summer to winter, the influence of vegetation and terrain weakens gradually, while that of climate and latitude strengthens gradually. By spring, the influence of these factors has changed dramatically, and is similar to that in summer. Altitude has the most powerful effect on winter TFC. For two-factor interactions, their explanatory abilities are higher than 50%. There are the strongest explanatory abilities of vegetation-altitude, vegetation-climate and altitude-latitude interactions. The vegetation-altitude interaction has the strongest explanatory effect, which can explain 61% of annual PE spatial variation, 64% of summer PE spatial variation and 46% of autumn PE spatial variation. Then the vegetation-climate interaction can explain 66% of spring PE spatial variation. Finally, the altitude-latitude interaction can explain 56% of winter PE spatial variation.

These results can help to provide insights into the influencing process of environmental factors on the climate system and complexity mechanisms of the climate system. They are also conducive for evaluating the predictability of climate change in different regions and understand the uncertainty sources of the climate prediction results. In this research, we carry out regional comparison of TFC within the research area. In the future, it is worthwhile to conduct the study with a larger spatial scale to compare TFC among China and other regions. The framework to detect the driving factors of complexity in this study can also be applied to other fields, like the fields of hydrology and ecology. In future research, it is recommended to analyze in depth the influence mechanism of driving factors on TFC. It is also recommended to predict the trend of regional TFC and complexity of regional climate systems.

## Supplementary Materials

The following are available online at https://www.mdpi.com/1099-4300/21/10/1001/s1.
