# Supplementary material for: Permutation Entropy-Based Analysis of Temperature Complexity Spatial-Temporal Variation and Its Driving Factors in China
Source: Entropy (Basel). 2019 Oct 13;21(10):1001. doi: 10.3390/e21101001 (PMC7514212; doi:10.3390/e21101001)
Supplement: Supplementary file 1 [file entropy-21-01001-s001.pdf]

Support information 1:

The spatial distribution of annual mean air temperature in China is plotted in Fig. S1a. The statistical histogram of annual and seasonal mean air temperature is described in Fig. S1b and Fig. S2 respectively. Table S1 records the regional average, regional maximum and regional minimum for both the annual and season temperature in China.

Seasonal mean temperature varies among different locations in China. Spring mean temperature ranges from -11.39 – 25.43°C in China, with more regions' temperature over 0°C (Fig S2a). Summer mean temperature ranges from -0.98 – 29.13°C in China. Autumn mean temperature ranges from -10.14 – 25.98°C in China. Winter mean temperature ranges from -25.50 – 20.54°C in China, with more regions' temperature below 0°C (Fig S2d).

The above statistics are derived from the mean daily temperature data from 1979 to 2017 in China.

Table 1. The regional statistical data on air temperatures.

Air Temperature (°C)	Regional Average	Regional Maximum	Regional Minimum
spring	9.32	25.43	−11.39
summer	20.16	29.13	−0.98
autumn	8.92	25.98	−10.14
winter	-4.59	20.54	−25.50
annual	8.51	25.20	−11.01

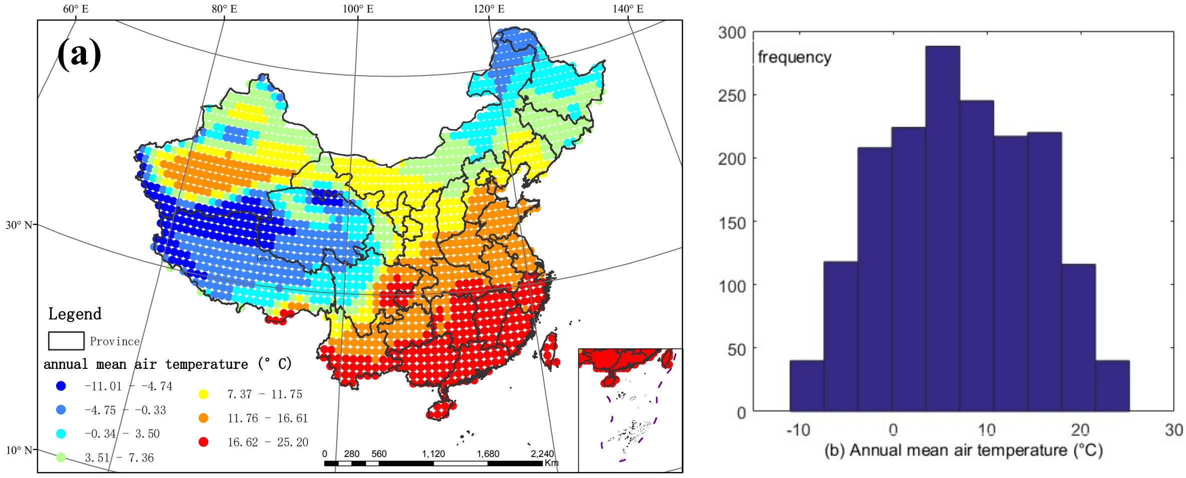

(a) (b)

Figure 1. (**a**) Spatial distribution of annual mean air temperature of China and (**b**) the statistical histogram of annual mean air temperature.

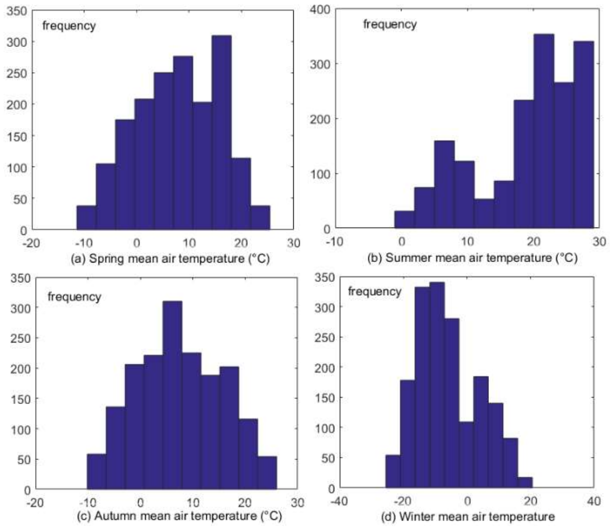

Figure 2. The statistical histogram of seasonal mean air temperature.

Support information 2:

The process of temperature change may be different for places with similar TFC.

For example, PE is similar in Xinjiang Basin and southeastern China, and the temperature fluctuation complexity in these areas is similar, but their actual probability distributions of temperature trends are different. We chose two pairs of locations in the Northwest and the Southeast with identical PE to compare their temperature change.

One permutation corresponds to one status of five-day temperature change. There are 120 (5!) permutations in total. For example, Permutation 1 represents that the temperature trend within five days is T5<T4<T3<T2<T1 (T*i* is the temperature in day *i*). Top 10 permutations and their probability were recorded in Table S2.

Table 2. The codes of Sub-regions for each driving factor.

**Experiment 1**										
Location 1 (Northwest): 41.25° N, 87.75° E PE= 0.8248										
Probability	0.124	0.046	0.040	0.037	0.030	0.029	0.025	0.024	0.024	0.023
Permutation	109	1	110	92	2	25	84	114	5	49
Location 2 (Southeast): 24° N, 110.25° E PE= 0.8248										
Probability	0.121	0.040	0.036	0.035	0.030	0.030	0.029	0.028	0.027	0.023
Permutation	109	92	1	110	84	2	5	114	25	26
**Experiment 2**										
Location 1 (Northwest): 41.25° N, 88.5° E PE= 0.8253										
Probability	0.124	0.041	0.038	0.036	0.030	0.029	0.026	0.025	0.025	0.024
Permutation	109	1	110	92	2	25	84	114	5	26
Location 2 (Southeast): 23.25° N, 111° E PE= 0.8255										
Probability	0.124	0.041	0.038	0.036	0.030	0.029	0.026	0.025	0.025	0.024
Permutation	109	92	1	110	2	84	5	25	114	26

Support information 3:

The average of annual and seasonal TFC intensity is calculated in each sub-region for all factors respectively. The results are recorded in the Table S4-S5 respectively.

Table 3. codes of Sub-regions for each driving factor.

Vegetation		Climate		Altitude		Latitude		Terrain	
1	Subtropical evergreen broad-leaved forest	1	Middle Temperature Zone	1	-263-565 meter	1	18.75-23.75°N	1	plains
2	Cold-temperate coniferous forest	2	Warm Temperate Zone	2	1566-1220 meter	2	24-28.5°N	2	platforms
3	Warm-temperate deciduous broad-leaved forest	3	Cold Temperate Zone	3	1221-2045 meter	3	29.25-34.5°N	3	hills
4	Temperate grassland	4	North Subtropical Zone	4	2046-3085 meter	4	35.25-39.75°N	4	small relief mountains
5	Temperate desert	5	Central Subtropical Zone	5	3086-4050 meter	5	40.5-45°N	5	medium relief mountains
6	Temperate coniferous and deciduous broad-leaved mixed forest	6	South Subtropical Zone	6	4051-4845 meter	6	45.75-51°N	6	large relief mountains
7	Tropical monsoon rainforest and rainforest area	7	Middle Tropical Zone	7	4846-8535 meter	7	51.75-53.25°N	7	extreme relief mountains
8	Alpine vegetation area	8	Marginal Tropical Zone						
		9	Plateau Climatic Zone						

Table 4. The annual TFC intensity of each sub-region for all factors.

Vegetation (37%)		Climate (16%)		Altitude (11%)		Latitude (9%)		Terrain (5%)	
5	0.850	7	0.839	2	0.861	1	0.846	3	0.863
1	0.861	6	0.847	1	0.865	2	0.860	1	0.865
8	0.880	8	0.864	7	0.874	5	0.866	2	0.868
2	0.879	2	0.861	4	0.873	4	0.869	4	0.871
4	0.881	4	0.865	5	0.880	6	0.872	5	0.874
7	0.871	5	0.860	3	0.867	3	0.875	6	0.876
3	0.882	1	0.872	6	0.883	7	0.877	7	0.882
6	0.886	3	0.878						
		9	0.879						

Table 5. The seasonal TFC intensity of each sub-region for all factors.

Spring									
Vegetation (47%)		Climate (16%)		Altitude (10%)		Latitude (9%)		Terrain (5%)	
5	0.823	7	0.805	2	0.845	1	0.828	3	0.847
1	0.857	6	0.832	3	0.851	5	0.852	1	0.856
7	0.860	2	0.837	4	0.858	4	0.853	2	0.860
8	0.870	8	0.849	7	0.863	2	0.856	4	0.865
4	0.879	5	0.854	1	0.865	3	0.869	5	0.865
2	0.880	4	0.865	5	0.873	6	0.870	6	0.867
3	0.881	1	0.865	6	0.875	7	0.879	7	0.879
6	0.892	9	0.869						
		3	0.880						
Summer									
Vegetation (45%)		Climate (10%)		Altitude (10%)		Latitude (4%)		Terrain (9%)	
5	0.857	2	0.873	2	0.875	5	0.882	3	0.877

1	0.884	4	0.876	3	0.885	1	0.882	1	0.880
2	0.893	6	0.880	1	0.886	4	0.882	2	0.885
8	0.899	5	0.882	4	0.886	2	0.887	4	0.892
4	0.900	7	0.885	7	0.896	7	0.888	5	0.897
7	0.904	1	0.889	5	0.899	6	0.889	6	0.901
3	0.907	3	0.892	6	0.904	3	0.896	7	0.907
6	0.910	9	0.898						
		8	0.901						
**Autumn**									
**Vegetation (24%)**		**Climate (12%)**		**Altitude (6%)**		**Latitude (8%)**		**Terrain (1%)**	
5	0.865	6	0.858	1	0.869	1	0.862	1	0.871
1	0.865	4	0.863	7	0.872	2	0.864	3	0.873
8	0.878	5	0.863	2	0.872	5	0.874	2	0.875
7	0.883	2	0.874	3	0.876	3	0.876	4	0.876
3	0.886	1	0.877	4	0.882	6	0.878	5	0.876
4	0.887	9	0.880	6	0.884	4	0.879	6	0.877
6	0.888	8	0.885	5	0.884	7	0.890	7	0.883
2	0.889	3	0.890						
		7	0.892						
**Winter**									
**Vegetation (20%)**		**Climate (28%)**		**Altitude (14%)**		**Latitude (27%)**		**Terrain (1%)**	
7	0.855	7	0.828	1	0.865	1	0.836	7	0.874
1	0.863	6	0.833	2	0.874	2	0.864	4	0.874
2	0.872	8	0.851	3	0.876	7	0.868	2	0.874
5	0.873	4	0.862	4	0.882	6	0.876	3	0.874
6	0.881	5	0.866	7	0.883	4	0.877	1	0.874
3	0.882	3	0.871	5	0.884	5	0.881	6	0.876
4	0.882	1	0.877	6	0.889	3	0.883	5	0.878
8	0.888	2	0.879						
		9	0.884						

Support information 4:

The series of annual TFC in Fig 9a of main text is the average of PE series at all locations. Nevertheless, such regional average may cover up the obvious spatial variation of temporal trends in different regions. Therefore the spatial K-means clustering was used to analyze the annual TFC trend of each spatial cluster from 1979-2017.

Spatial cluster analysis, as a multivariate statistical analysis method, can classify a non-classified labeled sample into several categories according to the similarity of their time series. It classifies similar samples into one cluster and divides them into different clusters as much as possible. K-means method is one of the most popular clustering methods. K-means clustering analysis is conducted in MATLAB Software by function called k-means clustering. It can be downloaded from (https://ww2.mathworks.cn/matlabcentral/fileexchange/71796-k-means-clustering). This function can be used directly to cluster n observations into k groups in which each point belongs to the cluster with the nearest mean. It can classify a large number of data efficiently according to its time series, but it needs to predetermine the number of clusters (k).

Here we use SSE (sum of the squared errors) to determine the reasonable number of clusters.

$$\mathrm{SSE} = \sum_{p \in C_i} |p - m_i|^2$$

where C_i corresponds to the Cluster i and p is the location point within this cluster. m_i is the cluster center of Cluster i.

The possible spatial cluster situations were demonstrated by K-means clustering, and SSE of all clustering results was calculated (Fig S3). When SSE descends slowly and achieves convergence, it

corresponds to the optimal number of cluster. The principle is as follows: with the increase of cluster number *k*, the sample division will be finer. Therefore the aggregation degree of each cluster will gradually increase, and the square of error and SSE will naturally become smaller. Moreover, when *k* is less than the optimal number of clusters, the decrease of SSE will be large because the increase of *k* will greatly increase the aggregation degree of each cluster. When *k* reaches the optimal number of clusters, the aggregation degree will only decrease slightly, so the decrease of SSE will slow sharply. Then SSE tends to flatten as the increase of *k*. *k* with such abrupt change of SSE corresponds to the best clustering number.

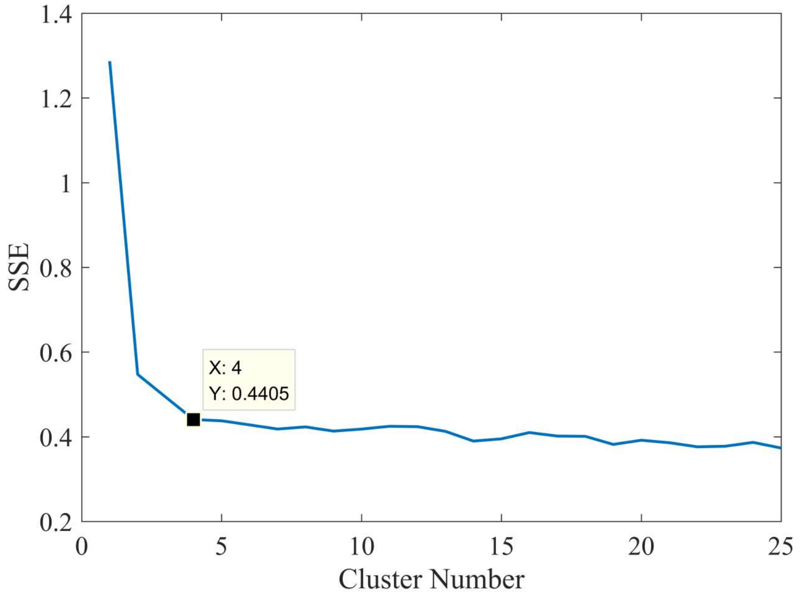

Figure 3. SSE of clusters with cluster number from 2–25.

From Figure S3, four is the optimal cluster number. The trend and mutation characteristics were analyzed for four clusters by Mann-Kendall method in Figure S4.

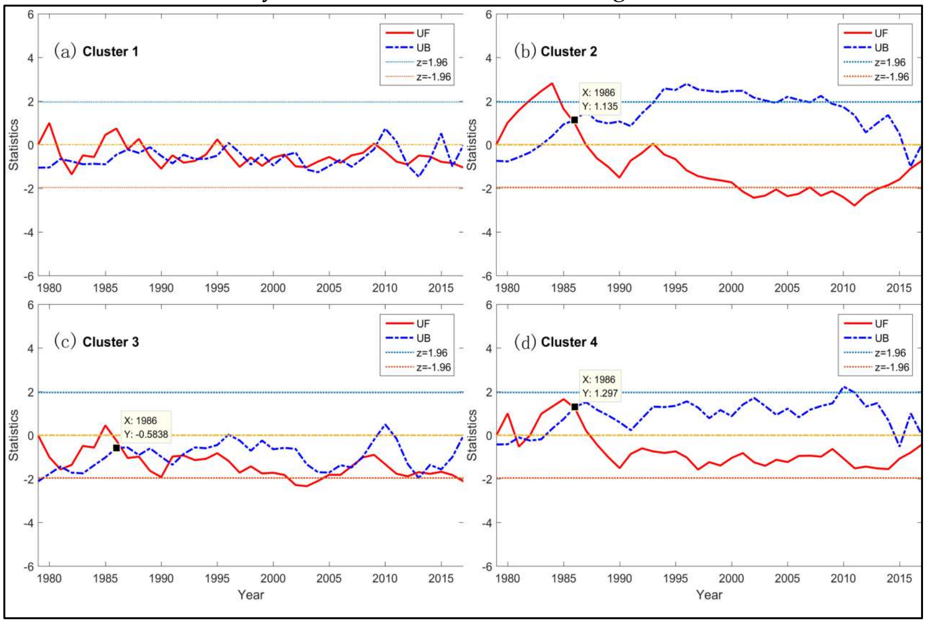

Figure 4. Temporal trend and mutation of annual TFC for four clusters.

Support information 5

Time series of seasonal temperature fluctuation complexity were depicted in Fig S5. The black dash line marked the lowest TFC intensity. In summer, autumn and winter, the seasonal TFC reached the lowest level in 2001, 2011 and 2012 respectively (Fig S5b-d). The weakest spring TFC occurred in 1990 and 2014 (Fig S5a).

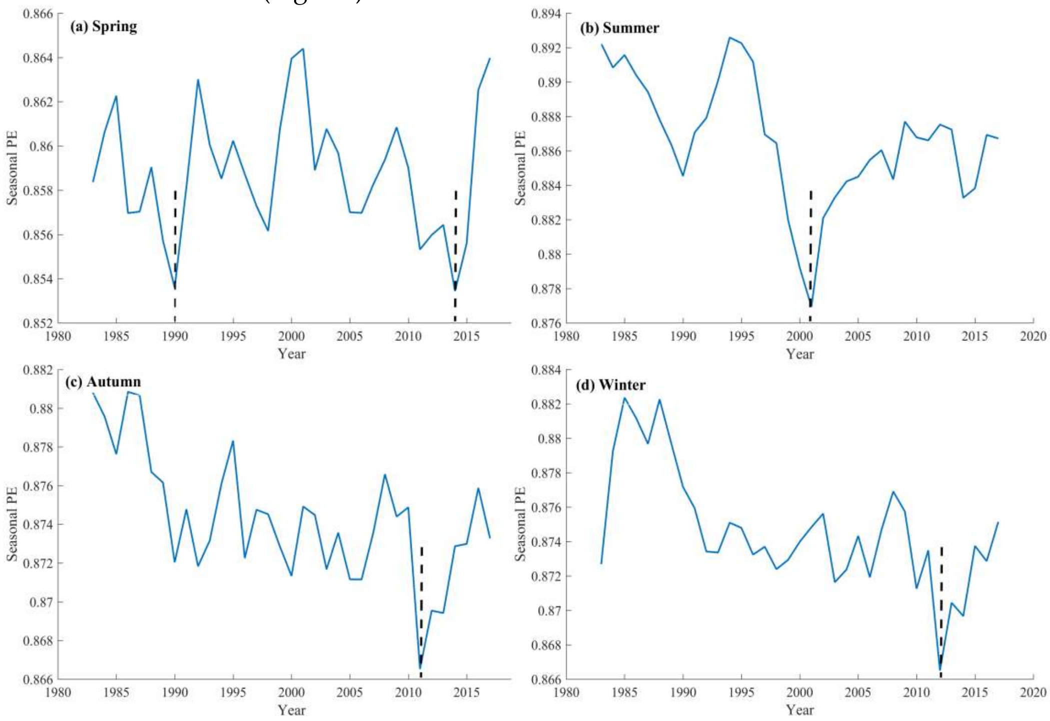

Figure S5. Time series of seasonal TFC.

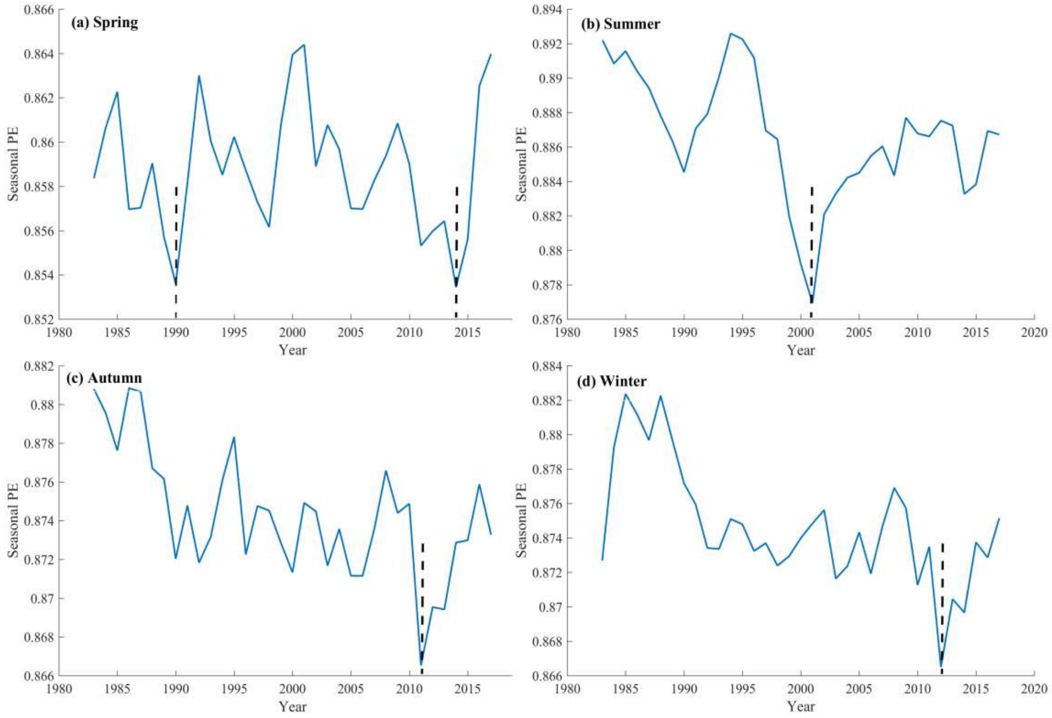

Figure S5. Time series of seasonal TFC

However the series of seasonal TFC in Figure S5 is also the average of PE series at all locations. Same as the clustering analysis in Support information 4, the spatial K-means clustering was also used to analyze the seasonal TFC trend of each spatial cluster from 1983-2017 in case that such regional average may cover up the obvious spatial variation of temporal trends in different regions. The specific clustering calculation process is conducted as the description in Support information 4.

The possible spatial cluster situations for each season were demonstrated by K-means clustering, and SSE of all clustering results was calculated (Fig S6). From Fig S6, five is found to be the optimal cluster number for each season and the spatial distribution of these five clusters in each season is described in Fig S7.

For each season, the trend in each cluster is similar to each other (Fig S8). The black dash line in Fig S8 marked the lowest TFC intensity. The results prove that the trend of seasonal TFC in all clusters is similar to that of average seasonal TFC. And the time of the lowest seasonal TFC for each cluster is identical with that for regional average series (Fig S5). For example, in spring, almost all clusters in China reached the weakest TFC in 1990 and 2014 (Fig S8a), which is the same as results in Fig S5a. In summer, autumn and winter, most clusters reached the lowest seasonal TFC in 2001, 2011 and 2012 respectively (Fig S8b-d). In general, the trend analysis of regional average of the seasonal TFC is representative.

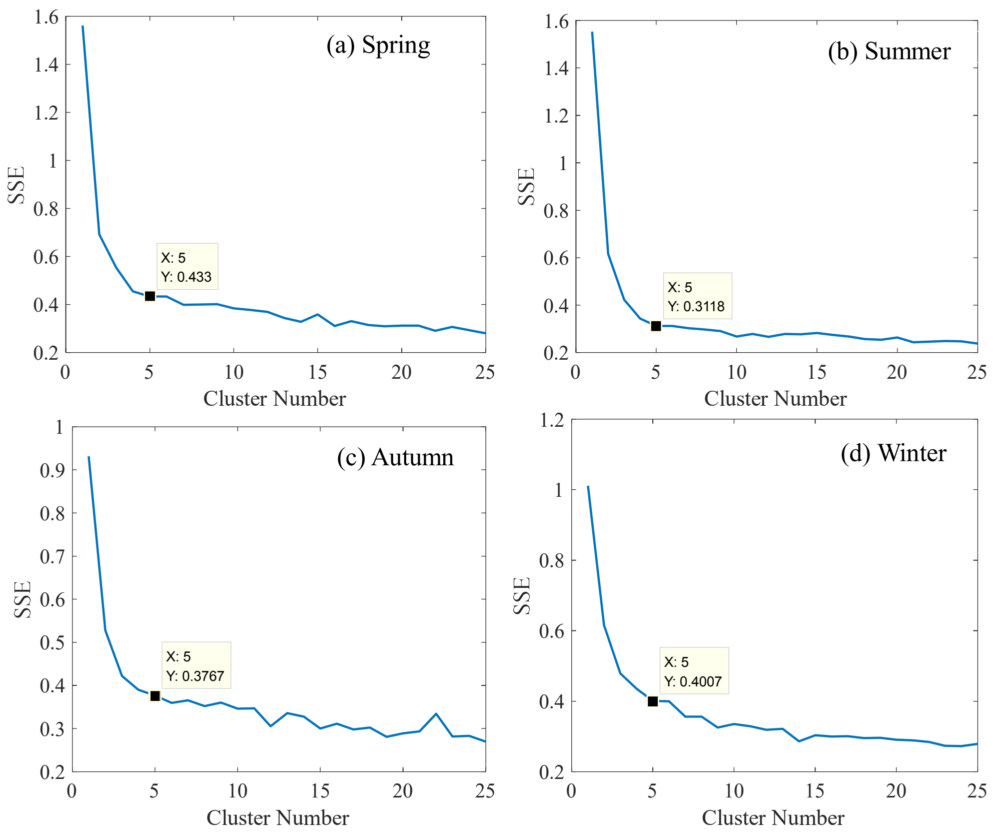

Figure S6. SSE of clusters with cluster number from 2-25 in each season

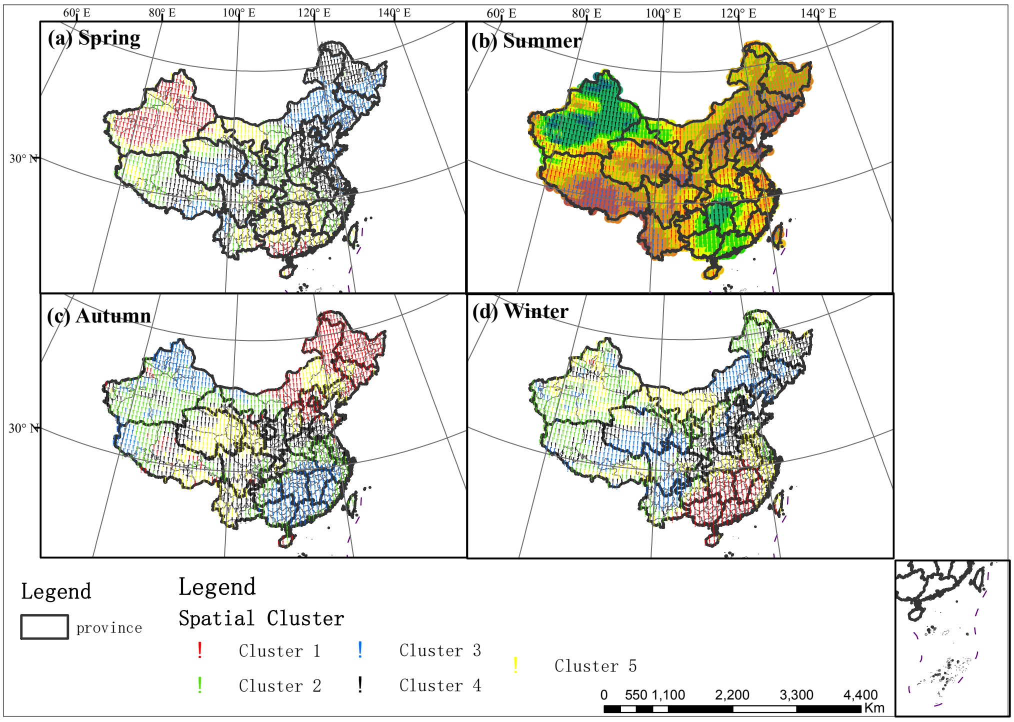

Figure S7. Spatial distribution of five TFC clusters in China for each season

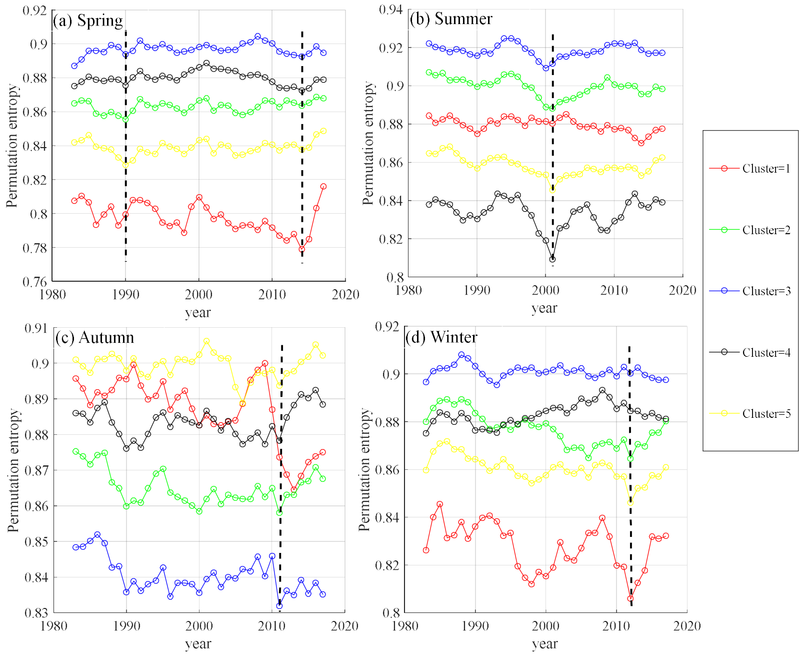

Figure S8. Time series of five TFC clusters in each season. The black dash line marked the lowest seasonal TFC intensity for each season.
